# Does the 12-item General Health Questionnaire contain multiple factors and do we need them?

**DOI:** 10.1186/1477-7525-2-63

**Published:** 2004-11-11

**Authors:** Fei Gao, Nan Luo, Julian Thumboo, Calvin Fones, Shu-Chuen Li, Yin-Bun Cheung

**Affiliations:** 1 Clinical Trials and Epidemiological Sciences, National Cancer Centre, 11 Hospital Drive, 169610, Singapore; 2 Institute of Health Economics, #1200, 10450 Jasper Avenue, Edmonton, Alberta, T5J 3N4, Canada; 3 Department of Rheumatology and Immunology, Singapore General Hospital, Outram Road, 169608, Singapore; 4 Department of Psychological Medicine, National University of Singapore, 5 Lower Kent Ridge Road, 119074, Singapore; 5 Department of Pharmacy, National University of Singapore, 18 Science Drive 4, 117543, Singapore; 6 MRC Tropical Epidemiology Group, IDEU, ITD, London School of Hygiene and Tropical Medicine, London WC1E 7HT, UK

**Keywords:** GHQ, factor structure, psychological health

## Abstract

**Background:**

The 12-item General Health Questionnaire (GHQ-12) is widely used as a unidimensional instrument, but factor analyses tended to suggest that it contains two or three factors. Not much is known about the usefulness of the GHQ-12 factors, if they exist, in revealing between-patient differences in clinical states and health-related quality of life.

**Methods:**

We addressed this issue in a cross-sectional survey of out-patients with psychological disorders in Singapore. The participants (n = 120) completed the GHQ-12, the Beck Anxiety Inventory, and the Short-Form 36 Health Survey. Confirmatory factor analysis was used to compare six previously proposed factor structures for the GHQ-12. Factor scores of the best-fitting model, as well as the overall GHQ-12 score, were assessed in relation to clinical and health-related quality of life variables.

**Results:**

The 3-factor model proposed by Graetz fitted the data better than a unidimensional model, two 2-factor models, and two other 3-factor models. However, the three factors were strongly correlated. Their values varied in a similar fashion in relation to clinical and health-related quality of life variables.

**Conclusions:**

The 12-item General Health Questionnaire contains three factors, namely Anxiety and Depression, Social Dysfunction, and Loss of Confidence. Nevertheless, using them separately does not offer many practical advantages in differentiating clinical groups or identifying association with clinical or health-related quality of life variables.

## Background

Recent studies of disease burden have demonstrated the importance of psychological disorders. For instance, depression was the fourth leading cause of disease burden, accounting for 4.4% of total disability adjusted life years in the world in 2000 [1]. The 12-item General Health Questionnaire (GHQ-12) has been widely used in many countries for detecting psychological morbidity. Some major national studies such as the British Household Panel Survey (BHPS) also employ this instrument [2]. Calibration of this instrument may therefore contribute significantly to a large community of researchers.

While the longer versions of the GHQ are normally considered multidimensional, the GHQ-12 is often regarded as measuring only a single dimension of psychological health. For example, Corti [3] analyzed the GHQ-12 data in the BHPS and maintained that the high Cronbach's alpha value indicated the unidimensionality of this instrument. However, several authors suggested that the GHQ-12 contained two or three clinically meaningful factors. Using principal component analysis, Politi et al. [4] identified two factors: general dysphoria and social dysfunction. Andrich and van Schoubroeck [5] suggested that the positively worded items formed one factor and the negatively worded items formed another. Graetz [6], Martin [7] and Worsely and Gribbin [8] proposed three different 3-factor models. In a multi-centre study, although considerable between-centre variation was found, the final solution tended to have either two or three factors [9].

Using confirmatory factor analysis (CFA) to analyze the BHPS data, Cheung [10] compared various models and found that the 3-factor model proposed by Graetz [6] gave the best fit. The factors are anxiety and depression (4 items), social dysfunction (6 items), and loss of confidence (2 items). In a study of employees in New Zealand, Kalliath et al [11] also employed CFA to compare various models. They also found that Graetz's 3-factor model gave better goodness-of-fit than the others. However, they maintained that none of the models they examined gave a sufficient level of goodness-of-fit. Hence they modified the instrument to propose a short (8-item) version of GHQ. In a study of college students and young adolescents in Australia, French and Tait [12] found that Graetz's model not only fitted the data better than other models, but also satisfactorily achieved some fit indices targets such as Comparative Fit Index > 0.95. In a study of a rural population in Australia [13], the model of Worsely and Gribbin fitted best and that of Graetz was second best.

While the structure of the GHQ-12 has been studied using factor analysis methods, the construct validity and usefulness of those resulting factors are not often tested. The question is whether the additional information provided by the 2 or 3 factors, if they exist, is clinically useful. In other words, will multiple scores be more useful than a total single score in helping us to understand respondents' health status?

The purpose of this study was therefore two-fold. First, we aimed to compare the previously proposed models of the GHQ-12 in an oriental population and identify the best-fitting one. It was not our objective to assess their absolute level of fit or to derive new model or version of the GHQ. Second, we aimed to assess whether the factors identified relate to clinical and health-related quality of life variables in different ways.

## Methods

### Subjects and study design

A consecutive sample of outpatients with anxiety disorders and/or depressive disorders was recruited from a psychiatric clinic at a tertiary hospital in Singapore. Inclusion criteria were the presence of any anxiety disorder and/or major depressive disorder, literacy in English or Chinese, and completion of an informed consent form. Patients with organic brain syndrome or psychosis were excluded.

During routine consultation visits, diagnoses of recruited patients were ascertained by a psychiatrist using DSM-IV criteria and the severity of their psychiatric disorders was assessed using a Clinical Global Impression (CGI) scale, which ranges from 1 (very mild) to 5 (very severe). Patients were then given a questionnaire containing the General Health Questionnaire (GHQ-12) [14], the Beck Anxiety Inventory (BAI) [15], and the Short Form-36 Health Survey (SF-36) [16] for self-completion. Identical English and Chinese questionnaires were prepared for subjects to select according to their preference. A research assistant checked returned questionnaires for completeness.

### Instruments

The General Health Questionnaire (GHQ-12) consists of 12 items, each assessing the severity of a mental problem over the past few weeks using a 4-point scale (from 0 to 3). The score was used to generate a total score ranging from 0 to 36, with higher scores indicating worse conditions [14]. The Chinese version of GHQ-12 used in this study had been validated [17,18]. A previous study of the 60- and 30-item versions of English and Chinese GHQ yielded comparable scale scores, suggesting equivalence for the two language versions [19].

The Beck Anxiety Inventory (BAI) is a valid and reliable self-report checklist for anxiety symptoms [15]. This instrument consists of 21 items, each describing an anxiety symptom for a respondent to assess how much he or she has been bothered by the symptom over the past week on a 4-point scale. Responses to all items are summed up to a total score ranging from 0 to 63, with higher scores indicating more severe anxiety. A Chinese BAI was developed by the authors using forward- and back-translation procedures, and refined after a pilot study of subjects with anxiety disorders [20].

The Short Form 36 Health Survey (SF-36) [16] is a 36-item questionnaire assessing functional health-related quality of life (HRQoL) in 8 domains: physical functioning, role limitations due to physical problems, bodily pain, general health, vitality, social functioning, role limitations due to emotional problems, and mental health. The instrument yields each domain a score ranging from 0 to 100, with higher scores indicating better HRQoL. The validity and reliability of SF-36 have been extensively documented [21]. In Singapore, both the UK English [16] and Chinese (Hong Kong) [22] versions of SF-36 have been validated [23,24] and these two language versions appear to be equivalent [25].

### Statistical analysis

Various factor structures of the GHQ-12 were tested by confirmatory factor analysis. Model I was unidimensional. Model IIA contained 2 factors: General Dysphoria and Social Dysfunction [4]. Model IIB also contained 2 factors: positively worded items forming one factor and negatively worded items forming another [5]. Model IIIA contained 3 factors: Cope, Stress and Depress, identified by Martin [7]. Model IIIB was the 3-factor model proposed by Graetz [6]: Anxiety and Depression, Social dysfunction, and Loss of Confidence. Model IIIC was also a 3-factor model: Anhedonia-Sleep disturbance, Social Performance and Loss of Confidence [8]. In the confirmatory factor analysis the number of factors and the relationship between factors and observed GHQ-12 items were pre-specified according to the models. The loading of an item on a factor within a model was estimated using the maximum likelihood method.

Methodologists have emphasized that it is desirable to use different indicators to examine a model's goodness-of-fit [26]. The fit of the six models was assessed by three measures. The Akaike's Information Criterion (AIC) penalizes the maximum log likelihood of a model according to its number of parameters. A model with a lower AIC is more plausible than one with a higher AIC. Instead of showing relative fitness, the Comparative Fit Index (CFI) assesses the fit of a model itself. The values range between 0 and 1. A CFI larger than 0.90 indicates an acceptable model. (Hu and Bentler [27] suggested that a CFI value above 0.95 indicates an acceptable model. In a later section we will discuss the more stringent cutoff.) The Root Mean Square of Approximation (RMSEA) assesses a model's amount of error. An RMSEA value larger than 0.08 indicates too much error.

The best-fitting model was examined in detail. The Kruskal-Wallis test was used to compare the GHQ-12 overall and factor scores of patients with different diagnosis. Pearson's correlation coefficient (r) was used to assess the association between GHQ-12 scores and various variables, namely Beck Anxiety Inventory, Clinical Global Impression and SF-36 scores. The Fisher's Z transformation was used to produce 95% confidence interval.

## Results and Discussion

A total of 120 participants (63 man and 57 women) were included in the analysis (Table 1). Most (90%) respondents were Chinese; the mean (SD) age was 43.1 (12.7). Sixty six percent of the participants chose to administer an English version of the questionnaire. The mean scores of clinical and HRQoL data reported by the respondents in both gender were shown in Table 1. Men tended to have less anxiety, better clinical global impression, and higher SF-36 scores.

**Table 1:** Mean (SD) clinical and SF-36 health-related quality of life values by gender

Clinical or psychological data	Men (*N *= 63)	Women (*N *= 57) ^(a)^
Beck Anxiety Inventory	20.65 (13.48)	21.89 (13.59)
Clinical Global Impression	2.76 (0.84)	3.02 (0.82)
Physical Functioning	76.50 (17.88)	73.72 (17.97)
Physical Problems	51.06 (43.57)	43.42 (42.13)
Bodily Pain	62.06 (24.89)	53.46 (24.05)
General Health	49.48 (21.14)	49.21 (20.07)
Vitality	45.40 (18.15)	41.26 (19.94)
Social Functioning	56.15 (23.75)	50.22 (26.88)
Emotional Problems	39.15 (42.56)	26.32 (41.18)
Mental Health	51.05 (17.65)	46.67 (19.11)

(a) N = 56 for Clinical Global Impression scale due to a missing value.

Table 2 shows goodness-of-fit statistics for the 1-, 2- and 3-factor models. The 3-factor model (IIIB) proposed by Graetz (1991) was the best in terms of all three fit statistics. It gave the lowest AIC and RMSEA and highest CFI. Its CFI was 0.935. All six models produced RMSEA's which exceeded 0.08. The one-dimensional model (Model I) had the highest AIC, highest RMSEA and lowest CFI.

**Table 2:** Goodness-of-fit of six confirmatory factor analysis models (N = 120) ^(a),(b)^

Statistics	Model I (1 factor)	Model IIA (2 factors)	Model IIB (2 factors)	Model IIIA (3 factors)	Model IIIB (3 factors)	Model IIIC (3 factors)
AIC	69.529	29.220	29.956	51.611	21.075	48.956
CFI	0.888	0.927	0.925	0.908	0.935	0.910
RMSEA	0.139	0.115	0.115	0.130	0.109	0.128

(a) AIC: Akaike's Information Criterion; CFI: Comparative Fit Index; RMSEA: Root Mean-Square Error of Approximation

(b) See text for details about the models.

**Figure 1:**
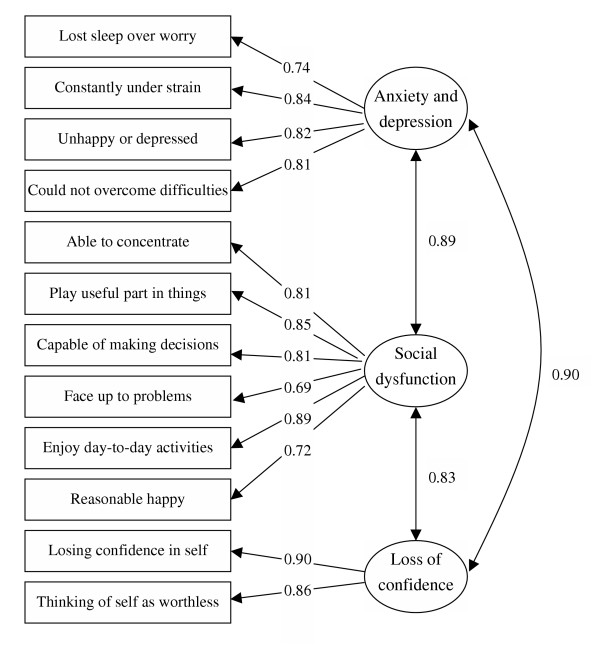
Standardised factor loadings and between-factor correlations of Graetz's model [6]. Boxes represent GHQ-12 items; ellipses represent factors. One-way and two-way arrows indicate factor loadings and between-factor correlations, respectively.

Figure 1 displays the standardized factor loadings and between-factor correlation of model IIIB. The factor loadings ranged between 0.72 and 0.90. The three factors were strongly correlated. The correlation between factor 1 (Anxiety and Depression) and factor 2 (Social Dysfunction) was 0.89. The correlation between factor 2 and factor 3 (Loss of Confidence) was 0.83. That between factor 1 and 3 was 0.90. These strong correlations suggest that even if there were in fact three factors, in practice it may be very difficult to discern them.

Having established that Graetz's 3-factor model fitted the data better than the other models, we calculated the factor scores as unweighted sums of the items concerned. From figure 1 we could see that the loadings on each factor did not vary substantially. Hence we chose to use unweighted sums for simplicity. Table 3 shows the mean (SD) factor scores and the overall GHQ-12 score by clinical diagnosis. Some patients had multiple diagnoses; we categorized them into one of three major clinical diagnoses. The three factor scores and the overall GHQ-12 scores behaved in fairly similar ways. All four scores were significantly different between patients with and without depression; none was significantly different between patients with and without general anxiety disorder. Patients with panic disorder had lower scores on the factor Loss of Confidence (difference = 0.68; P = 0.043). The SD of the two diagnosis groups pooled was about 1.75; the between group difference was therefore approximately about 0.4 SD.

**Table 3:** Comparison of mean (SD) values of GHQ-12 scores by clinical diagnosis.

Diagnosis	N	Overall GHQ-12 score	Anxiety and depression (Factor 1)	Social dysfunction (Factor 2)	Loss of confidence (Factor 3)
Depression					
Yes	60	32.15 (9.18)	11.28 (3.10)	15.73 (4.74)	5.13 (1.83)
No (Other diagnosis)	60	27.43 (6.74)	9.48 (2.86)	13.73 (3.06)	4.22 (1.62)
P-value ^(a)^		0.002	0.002	0.010	0.005
General anxiety disorder					
Yes	47	30.02 (8.10)	10.68 (2.99)	14.64 (3.96)	4.70 (1.82)
No (Other diagnosis)	73	29.64 (8.58)	10.19 (3.18)	14.79 (4.20)	4.66 (1.77)
P-value		0.712	0.410	0.987	0.993
Panic disorder					
Yes	54	28.48 (7.88)	9.96 (3.08)	14.22 (3.71)	4.30 (1.69)
No (Other diagnosis)	66	30.86 (8.64)	10.73 (3.10)	15.15 (4.37)	4.98 (1.80)
P-value		0.158	0.179	0.244	0.043

(a) Kruskal-Wallis test.

Table 4 presents the results of the correlation of 3 factors of Graetz's model and BAI, Clinical Global Impression Score, and SF-36 scales. The 3 factors were correlated with the 10 clinical and HRQoL variables to very similar degree.

**Table 4:** Pearson's correlation coefficients (95% confidence intervals) between GHQ-12 scores and clinical and health-related quality of life variables

Clinical/HRQoL scales	Overall GHQ-12 score	Anxiety and depression (Factor I)	Social dysfunction (Factor II)	Loss of confidence (Factor III)
Beck Anxiety Inventory	0.69 (0.58 to 0.77)	0.68 (0.57 to 0.77)	0.62 (0.50 to 0.72)	0.63 (0.50 to 0.72)
Clinical Global Impression	0.49 (0.34 to 0.61)	0.45 (0.29 to 0.58)	0.47 (0.31 to 0.60)	0.43 (0.27 to 0.56)
Physical functioning	-0.17 (-0.34 to 0.01)	-0.18 (-0.35 to 0.00)	-0.16 (-0.33 to 0.02)	-0.12 (-0.29 to 0.06)
Role physical	-0.63 (-0.73 to -0.51)	-0.60 (-0.71 to -0.48)	-0.61 (-0.71 to -0.48)	-0.51 (-0.63 to -0.36)
Bodily pain	-0.52 (-0.68 to -0.44)	-0.57 (-0.68 to -0.44)	-0.43 (-0.56 to -0.27)	-0.46 (-0.59 to -0.31)
General health	-0.57 (-0.68 to -0.44)	-0.57 (-0.68 to -0.43)	-0.52 (-0.64 to -0.38)	-0.50 (-0.62 to -0.35)
Vitality	-0.71 (-0.79 to -0.61)	-0.73 (-0.80 to -0.63)	-0.63 (-0.73 to -0.51)	-0.62 (-0.72 to -0.50)
Social functioning	-0.65 (-0.74 to -0.54)	-0.62 (-0.72 to -0.50)	-0.60 (-0.70 to -0.47)	-0.59 (-0.70 to -0.46)
Role emotional	-0.62 (-0.72 to -0.50)	-0.63 (-0.73 to -0.51)	-0.55 (-0.66 to -0.41)	-0.55 (-0.66 to -0.41)
Mental health	-0.67 (-0.76 to -0.56)	-0.67 (-0.76 to -0.56)	-0.60 (-0.70 to -0.47)	-0.61 (-0.71 to -0.49)

Several previous confirmatory factor analyses found that the 3-factor model of Graetz gave better fit to survey data from Australia [12], Britain [10] and New Zealand [11]. In this study we examined the issue in an Asian population in Singapore, whose members are mainly ethnic Chinese. All three goodness-of-fit indices employed, namely AIC, CFI and RMSEA, agreed that the 3-factor model of Graetz out-performed the other five models. The CFI value was 0.935. Conventionally, a CFI of 0.90 or larger is taken as evidence of sufficient fit. A more stringent criterion of CFI larger than 0.95 has recently been proposed and debated [27,28]. The RMSEA also indicated that even the best-fitting model did not fit well, using the cut-off of 0.08 as a criterion. However, our aim is to compare the models rather than to modify the instrument. So for our purpose it is the comparison of the goodness-of-fit of the six models that matters, not the absolute values of the fit indices. We consider the "correctness" and "usefulness" of a model two fairly separate issues. Although the goodness-of-fit of Graetz's model was limited, we proceeded to examine the factor scores in relation to external criteria in order to reach a conclusion about the usefulness of the model.

The one-dimensional model was the worst according to all three goodness-of-fit indices.

The three factors in the model proposed by Graetz were found to be strongly correlated with each other, with correlation coefficients in the neighborhood of 0.8 to 0.9. Such strong correlations suggest that even if there were indeed three different factors, in practice it is quite difficult to differentiate them. The study of French and Tait [12] also showed strong correlation between the factors, which led the authors to recommend that it may be prudent to use the overall score rather than overinterpret the factors within the GHQ-12. We examined the three factor scores and the overall GHQ-12 score in relation to clinical diagnoses. The four scores behaved in fairly similar ways. Although the Loss of Confidence scale was significantly different between patients with and without panic disorder while the other three scales did not show significant differences between the two groups of patients, the difference was only about 0.4 SD. This is smaller than a recommended threshold (0.5 SD) corresponding to minimal clinically important differences for health states questionnaires [29]. We also examined the association between the three GHQ scores and the Beck Anxiety Inventory, a clinical impression score, and the 8 scales of the SF-36. The three factors were associated with the clinical and HRQoL variables to similar degrees.

Two limitations of the study should be noted. Firstly, the sample size was somewhat small for confirmatory factor analysis. Secondly, the participants were clinical cases. This homogeneity might have made it more difficult to detect variations in GHQ-12 scores. We believe that the question about the relative plausibility of various factor models have been sufficiently answered by this and several previous studies [10-12]. Nevertheless, future studies of non-clinical participants based on larger sample sizes will be helpful to further assess the practical usefulness of the factors of the GHQ-12.

## Conclusions

Several studies, including the present one, have found that Graetz's 3-factor model of the GHQ-12 is more plausible than other models. However, the factors were strongly correlated and difficult to discern. Our analysis of the three GHQ scores in relation to clinical variables and aspects of health-related quality of life did not appear to be more informative than analysis of a single overall GHQ-12 score. As such, from a pragmatic point of view we consider it acceptable to use this instrument as a one-dimensional measure. Unless one has specific questions that are best answered by a subset of the three factors, there is no need to consider the multi-dimensionality.

## Authors' contribution

FG carried out the confirmatory factor analysis, interpreted the findings, and drafted part of the manuscript. NL designed the study, participated in the development of the statistical framework and interpreted the findings. JT participated in the study design, discussion of the statistical framework, and the interpretation of findings. CF participated in the study design and carried out the data collection and clinical assessments. SCL participated in the study design and discussion and interpretation of findings. YBC conceived of the study, developed the statistical framework, carried out part of the statistical analysis, and drafted part of the manuscript. All authors read and approved the final manuscript.

## References

[B1] Ustun TB, Ayuso-Mateos JL, Chatterji S, Mathers C, Murray CJ (2004). Global burden of depressive disorders in the year 2000. Br J Psychiatry.

[B2] Wiggins RD, Schofield P, Sacker A, Head J, Bartley M (2004). Social position and minor psychiatric morbidity over time in the British Household Panel Survey 1991–1998. J Epidemiol Community Health.

[B3] Corti L, Buck N, Gershuny J, Rose D, Scott J (1994). For better or worse? Annual change in smoking, self-assessed health and subjective well-being. In Changing Households: The British Household Panel Survey 1990–1992.

[B4] Politi PL, Piccinelli M, Wilkinson G (1994). Reliability, validity and factor structure of the 12-item General Health Questionnaire among young males in Italy. Acta Psychiatr Scand.

[B5] Andrich D, van Schoubroeck L (1989). The General Health Questionnaire: a psychometric analysis using latent trait theory. Psychol Med.

[B6] Graetz B (1991). Multidimensional properties of the General Health Questionnaire. Soc Psychiatry Psychiatr Epidemiol.

[B7] Martin AJ (1999). Assessing the multidimensionality of the 12-item General Health Questionnaire. Psychol Rep.

[B8] Worsley A, Gribbin CC (1977). A factor analytic study of the twelve-item General Health Questionnaire. Aust N Z J Psychiatry.

[B9] Werneke U, Goldberg DP, Yalcin I, Ustun BT (2000). The stability of the factor structure of the General Health Questionnaire. Psy Med.

[B10] Cheung YB (2002). A confirmatory factor analysis of the 12-item General Health Questionnaire among older people. Int J Geriatr Psychiatry.

[B11] Kalliath TJ, O'Driscoll MP, Brough P (2004). A confirmatory factor analysis of the General Health Questionnaire-12. Stress and Health.

[B12] French DJ, Tait RJ (2004). Measurement invariance in the General Health Questionnaire-12 in young Australian adolescents. Eur Child Adolesc Psychiatry.

[B13] Campbell A, Walker J, Farrell G (2003). Confirmatory factor analysis of the GHQ-12: can I see that again?. Aust N Z J Psychiatry.

[B14] Goldberg DP, Williams P (1988). A User's Guide to the General Health Questionnaire.

[B15] Beck AT, Epstein N, Brown G, Steer RA (1988). An inventory for measuring clinical anxiety: psychometric properties. J Consult Clin Psychol.

[B16] Ware JE, Snow KK, Kosinski M, Gandek B (1993). SF-36 Health Survey Manual and Interpretation Guide.

[B17] Chan DW, Chan TS (1983). Reliability, validity and the structure of the General Health Questionnaire in a Chinese context. Psychol Med.

[B18] Pan PC, Goldberg DP (1990). A comparison of the validity of GHQ-12 and CHQ-12 in Chinese primary care patients in Manchester. Psychol Med.

[B19] Chan DW (1985). The Chinese version of the General Health Questionnaire: does language make a difference?. Psychol Med.

[B20] Luo N, Fones CS, Thumboo J, Li SC (2004). Factors influencing health-related quality of life of Asians with anxiety disorders in Singapore. Qual Life Res.

[B21] McHorney CA, Ware JE, Lu JF, Sherbourne CD (1994). The MOS 36-item Short-Form Health Survey (SF-36): III. Tests of data quality, scaling assumptions, and reliability across diverse patient groups. Med Care.

[B22] Lam CLK, Gandek B, Ren XS, Chan MS (1998). Tests of scaling assumptions and construct validity of the Chinese (HK) version of the SF-36 Health Survey. J Clin Epidemiol.

[B23] Thumboo J, Feng PH, Soh CH, Boey ML, Thio ST, Fong KY (2000). Validation of the Chinese SF-36 for quality of life assessment in patients with systemic lupus erythematosus. Lupus.

[B24] Thumboo J, Fong KY, Machin D, Chan SP, Leong KH, Feng PH, Thio ST, Boe ML (2001). A community based study of scaling assumptions and construct validity of the English (UK) and Chinese (HK) SF-36 in Singapore. Qual Life Res.

[B25] Thumboo J, Fong KY, Chan SP, Machin D, Feng PH, Thio ST, Boey ML (2002). The equivalence of English and Chinese SF-36 versions in bilingual Singapore Chinese. Qual Life Res.

[B26] Marsh HW, Balla JR, Hau KT, Marcoulides GA, Schumacker RE (1996). An evaluation of incremental fit indices: a clarification of mathematical and empirical properties. In Advanced Structural Equation Modeling: Issues and Techniques.

[B27] Hu L, Bentler P (1999). Cutoff criteria for fit indexes in covariance structure analysis: conventional criteria versus new alternatives. Structural Equation Modeling.

[B28] Marsh HW, Hau KT, Wen Z (2004). In search of golden rules: comment on hypothesis-testing approaches to setting cutoff values for fit indexes and dangers in overgeneralizing Hu and Bentler's (1999) findings. Structural Equation Modeling.

[B29] Norman GR, Sloan JA, Wyrwich KW (2003). Interpretation of changes in health-related quality of life: the remarkable universality of half a standard deviation. Med Care.

